# A Response Surface Methodology (RSM) Approach for Optimizing the Attenuation of Human IgE-Reactivity to β-Lactoglobulin (β-Lg) by Hydrostatic High Pressure Processing

**DOI:** 10.3390/foods10081741

**Published:** 2021-07-28

**Authors:** Xin Sun, Jialing Vivien Chua, Quynh Anh Le, Francisco J. Trujillo, Mi-Hwa Oh, Dianne E. Campbell, Sam Mehr, Nanju Alice Lee

**Affiliations:** 1 School of Chemical Engineering, University of New South Wales, Syndey, NSW 2052, Australia; xinsun85@gmail.com (X.S.); vivien.j.chua@gmail.com (J.V.C.); quynh.le@unsw.edu.au (Q.A.L.); francisco.trujillo@unsw.edu.au (F.J.T.); 2 National Institute of Animal Science, Rural Development Administration, Wanju 55365, Korea; moh@korea.kr; 3 Immunology and Allergy, The Children’s Hospital at Westmead, Westmead, NSW 2145, Australia; dianne.campbell1@health.nsw.gov.au (D.E.C.); sam.mehr@me.com (S.M.)

**Keywords:** β-lactoglobulin, α-lactalbumin, high hydrostatic pressure processing, response surface models, rabbit IgG binding, human IgE-binding

## Abstract

The response surface methodology (RSM) and central composite design (CCD) technique were used to optimize the three key process parameters (i.e., pressure, temperature and holding time) of the high-hydrostatic-pressure (HHP) processing either standalone or combined with moderate thermal processing to modulate molecular structures of β-lactoglobulin (β-Lg) and α-lactalbumin (α-La) with reduced human IgE-reactivity. The RSM model derived for HHP-induced molecular changes of β-Lg determined immunochemically showed that temperature (temp), pressure (p^2^) and the interaction between temperature and time (*t*) had statistically significant effects (*p* < 0.05). The optimal condition defined as minimum (β-Lg specific) IgG-binding derived from the model was 505 MPa at 56 °C with a holding time of 102 min (R^2^ of 0.81 and *p*-value of 0.01). The validation carried at the optimal condition and its surrounding region showed that the model to be underestimating the β-Lg structure modification. The molecular change of β-Lg was directly correlated with HHP-induced dimerization in this study, which followed a quadratic equation. The β-Lg dimers also resulted in the undetectable human IgE-binding.

## 1. Introduction

Cow’s milk is one of the most common triggers of IgE-mediated food allergies in children and causes of food anaphylaxis deaths in young children [1]. One of the factors thought to contribute to the high prevalence in infants and children is their higher gastric pH of 3–4, forming unfavorable conditions for effective peptic hydrolysis when compared with an adult digestive system of pH 1.2–2 [2]. β-Lactoglobulin (β-Lg) and α-lactalbumin (α-La) are the major allergens in whey protein for which 60–80% of cow’s milk allergic patients react to [3,4]. β-Lg is highly stable to denaturation and resistant to proteolytic hydrolysis [5], whereas α-La is more thermally resistant than β-Lg [6,7]. Due to slow or poor degradation, β-Lg is thought to be absorbed through the gut mucosa as an intact molecule or as substantial fragments [2,8,9]. These proposed mechanisms could potentially induce sensitization in high-risk individuals.

Food processing has the potential to alter the allergenic properties of proteins by either masking, destroying or disclosing allergenic epitopes through cleavage of linear structures or inducing conformational changes, or by improving the digestibility of proteins. High hydrostatic pressure (HHP) processing could modify the molecular structure and functional properties of proteins and other associated macromolecules without affecting color, flavor and micronutrient contents [10]. For example, dissociation of oligomeric proteins and multi-protein complexes could be induced by the moderately high pressure of 100–200 MPa [11], whereas pressures of 400–800 MPa are sufficient to induce denaturation of small monomeric proteins [12]. Pressures between 200 and 500 MPa induce non-native monomers, dimers, trimers, and tetramers, while pressures >500 MPa induce higher molecular weight aggregates [13]. The typical pressure needed for the unfolding is around 500 MPa, however, it varies from protein to protein [14].

HHP induced immunoreactivity changes in food allergens have been demonstrated, but their reporting effects vary widely depending on the type of allergens, their antibody-binding epitopes and other extrinsic factors, such as processing conditions, single protein study vs mixed protein study, food matrix effects and characteristics of antibodies used for determining immunoreactivity. For example, the high pressure-induced conformational changes in the pork batter’s protein with the alteration of some epitope structures were reported [15]. In this study, the HHP at 600 MPa effectively modified the IgE immunoreactivity of the proteins in the sausage batter. The almond milk treated with high-pressure processing also showed complete elimination of conformation epitope and partial effects on linear epitopes [16]. The effect of HHP on β-Lg was also reported, which claimed an increased β-Lg immunoreactivity in whey, sweet whey and raw skim milk (200–600 MPa, 25–68 °C) [17].

Amongst structurally similar allergens, the effects of HHP could vary markedly. Of six nuts (pistachio, cashew, peanut, hazelnut, almond, and chestnut) treated with HHP (300–600 MPa, 20 °C, 20 min), only the immunoreactivities of pistachio, cashew, peanut and chestnut were altered [18]. The HHP-resistant property of almond allergens was also reported by Li et al. [19]. Even the same allergen could respond differently in different HHP studies, and this could be due to the experimental conditions as well as HHP equipment used. For example, the apple allergens, Mal d 1 and Mal d 1 responded to HHP by reducing their immunoreactivities in one study [20] and yet no effects were reported in another study [21]. Also, HHP resistant nature of fish allergens (Gad m 1 and largemouth bass) were reported with limited influence on their protein structures and immunoreactivities [22,23].

As a result of HHP-induced molecular changes, HHP also enhanced the digestibility of whey protein, which subsequently generates peptides that improve glutathione status in CFTR-deficient lung epithelial cells [24]. Employing high-pressure pretreatment for destabilizing milk proteins has been shown to enhance their susceptibility to enzymatic hydrolysis [24,25,26,27,28,29]. The proteolytic susceptibility of β-Lg increased 60- to 80-fold in the pressure range of 0.1–250 MPa [30]. Pressures ranging from 350 to 600 MPa dramatically increase the susceptibility of β-Lg to hydrolysis by pepsin, trypsin, chymotrypsin, pronase and thermolysin [31]. 

The HHP-enzymatic hydrolysis (EH) approach also improved the rate of hydrolysis at a pressure below 400 MPa [27,31,32,33,34,35]. HHP-EH induced bioactive peptides are another innovation. In comparison to EH, the HHP-EH process that combines pretreated protein hydrolysis with instantaneous pressurization and hydrolysis alters the peptide profiles in the hydrolysate. The process could also form novel peptides with increasing yield [29,36,37]. For example, by using chymotrypsin [36] and trypsin [29], HHP pretreated β-Lg marked by higher hydrophobicity. An elevation in bioactivity such as the antioxidant capacity of HHP-EH-generated peptides was also observed [35]. This is because HHP-EH generates higher low molecular weight peptides (<10 kDa) with lower immunoreactivity and higher digestibility [31,38].

Several limitations/drawbacks of HHP such as inactivation of enzymes and protein aggregation resulting from decreased protein solubility were also noted. A decreased hydrolysis by the inactivation of enzymes at elevated pressure could occur [27,37]. Protein aggregation also can arise during pressurization and decompression [39]. It has been suggested that hydrolysis should be implemented immediately after the HHP treatment to avoid the loss of susceptibility of HHP-treated protein to proteolysis over time [28]. Yet the long-term stability of HHP-treated proteins has not been explored.

As shown above, studies have shown the potential of HHP processing to induce molecular changes in protein with modified immunoreactivity, improved hydrolysis and digestibility, yet the optimum HHP processing conditions have not been modeled for whey protein targeting molecular changes with reduced (human) IgE reactivity. Therefore, in this study, the effects of three key process variables of HHP namely pressure, temperature and holding time were evaluated using a central composite design and modeled using the response surface methodology (RSM). The study determined optimum processing conditions defined as low IgG- and IgE-binding for whey protein allergens, β-Lg and α-La. Consequently, the correlation between rabbit IgG and human IgE-based immunoreactivity and dimerization was also investigated.

## 2. Materials and Methods

### 2.1. Materials

Whey protein and skim milk were prepared from raw milk, which was collected and delivered from the Lidcombe laboratory of Parmalat (Sydney, Australia). β-Lg A and β-Lg B from bovine milk, α-La from bovine milk, sodium dodecyl sulfate (SDS), 2-mercaptoethanol, goat anti-rabbit IgG-horseradish peroxidase (IgG-HRP), 3,3′,5,5′-Tetramethylbenzidine (TMB), Tween 20 (polyoxyethylene sorbitan monolaurate), sodium acetate (CH_3_COONa), beta-cyclodextrin and urea hydrogen peroxide, dimethylsulfoxide (DMSO)-99.9% molecular biology grade, sodium periodate, horseradish peroxidase (HRP), Bicinchoninic protein assay kit (BCA-1) containing bicinchoninic acid, sodium carbonate, sodium tartarate, sodium bicarbonate in 0.1 M NaOH (pH 11.25), copper (II) sulfate pentahydrate 4% (*w*/*v*) solution and a protein standard (i.e., bovine serum albumin(BSA)) with a concentration of 1.0 mg mL^−1^ were purchased from Sigma-Aldrich (St. Louis, MO, USA). Precision Plus Protein™ Prestained Standards (BioRad^®^, Hercules, CA, USA) ranging from 2 kDa to 250 kDa (161-0377), 40% acrylamide/bis solution (37.5:1) (161-0148), 2X Laemmli sample buffer (161-0737), 10x tris/glycine/SDS running buffer (161-0772), Tris (161-0719) were obtained from BioRad. Sodium chloride (NaCl), sodium hydrogen orthophosphate (NaH_2_PO_4_), disodium hydrogen orthophosphate (Na_2_HPO_4_), sodium bicarbonate (NaHCO_3_), sodium carbonate (Na_2_CO_3_), methanol, were purchased from Ajax Finechem (Seven Hills, NSW, Australia). Coomassie Brilliant Blue R-250 was purchased from Fluka (Loughborough, UK). Acetic acid glacial was from the Rhone Poulenc Laboratory Products (Clayton, VIC, Australia). NaH_2_PO_4_·2H_2_O was purchased from BDH Chemicals (Australia) Pty Ltd. (Port Fairy, VIC, Australia). Maxisorp polystyrene 96-well microwell plates were supplied by Nunc (Roskilde, Denmark).

### 2.2. Instrument

A Mini Protean Tetra Cell, power pack HC was purchased from BioRad. Microcentrifuge tubes were purchased from Eppendorf (Macquarie Park, Australia, state abbrev if appropriate, country). The SpectraMax M2, multi-detection microplate reader and SoftMax analysis software were from Molecular Devices (Sunnyvale, CA, USA). The pH Meter was supplied by TPS Pty Ltd. (Brisbane, Australia). A ChemiDoc MP system from BioRad was used for gel image analysis and dimerization semi-qualitification. A Stansted Food Processor S-FL-850-09-W was purchased from Stasted Fluid Power Ltd. (Harlow, Essex, UK). A Lauda Proline RP-890 thermostatic bath/circulator was purchased from LAUDA-Brinkmann, LP (Delran, NJ, USA).

### 2.3. HHP Treatments

β-Lg and α-La solutions at 300 μg mL^−1^ were added to screw-capped cryogenic tubes and the tubes were vacuum-sealed in the polyethylene bags. The HHP treatment was then performed using the Stansted S-FL-850-09-W Food Processor coupled with a thermostatic bath/circulator for the temperature control operated at conditions as listed in Table 1. The rates of pressurization and decompression were 200 MPa per min and the pressure-transmitting medium was 80% polypropylene glycol. Experiments were conducted in triplicates with PBS (0.1 M phosphate buffered saline, pH 7.4) and untreated protein solutions as the controls. After HHP treatment, samples were rested at 4 °C for 5 days before SDS-PAGE, ELISA, and BCA protein assay were performed. The 5-days resting period allowed the protein structures to come to stability and reduce deviation in subsequent measurements. The preliminary study of HHP conditions on the β-Lg IgG binding was conducted to determine the appropriate ranges of key operating parameters involving pressure and holding time required for a factorial experimental design.

### 2.4. Experimental Design and Data Analysis

A central composite design with three levels was performed to study the effects of the operating variables on the IgG binding and dimerization. As shown in Table 1, a CCD in the form of 3^3^ factorial design was used, in which three independent variables, pressure, temperature and holding time, were converted to respective dimensionless variables, x_1_, x_2_ and x_3_, and coded at 3 levels: −1, 0, +1. The parameter range was based on the preliminary study showing that the holding time does not need to be longer than 3 h because the pressure-induced changes occur rapidly, particularly at higher pressures. The temperature ranging from 11 to 78 ºC was chosen to cover from cooling to commercial pasteurization temperatures. The centre points were repeated 6 times to estimate the variability of the data. Each experiment was run in triplicates and the experimental order was randomized. The data obtained from the CCD was then fitted to a second-order polynomial regression equation (Equation (1)):

(1) $$\hat{y}=\hat{\beta_{0}}+\sum_{i=1}^{k}\hat{\beta_{i}}x_{i}+\sum_{i=1}^{k}\hat{\beta_{ii}}x_{i}^{2}+\sum_{i<j}\sum \hat{\beta_{ij}}x_{i}x_{j}\;,$$

### 2.5. Determination of Optimal Operating Conditions and Validation of the Model

When the contour plot displays ellipses or circles, the center of the system refers to a point of minimum responses called a saddle point. Those figures give useful information about the model showing general trends as explained by the fitted model but they may not represent the true behavior of the system. The minimum (or maximum) point occurs where the first derivative of the function equals zero.

(2) $$y=\hat{\beta_{0}}+\sum_{i=1}^{k}\hat{\beta_{i}}x_{i}+\sum_{i=1}^{k}\hat{\beta_{ii}}x_{i}^{2}+\sum_{i<j}^{}\sum \hat{\beta_{ij}}x_{i}x_{j}+\varepsilon ,$$

The minimum point is found by calculating $\frac{\partial y}{\partial x_{1}}$,$\;\frac{\partial y}{\partial x_{2}}$, and $\frac{\partial y}{\partial x_{3}}$, and set to zero (Equation (2)):

(3) $$\frac{\partial y}{\partial x_{1}}=\beta_{1}+2\beta_{4}x_{1}\;+\beta_{7}x_{2}+\beta_{8}x_{3}=0,$$

(4) $$\frac{\partial y}{\partial x_{2}}=\beta_{2}+2\beta_{5}x_{2}+\beta_{7}x_{1}\;+\beta_{9}x_{3}=0,$$

(5) $$\frac{\partial y}{\partial x_{3}}=\beta_{3}+2\beta_{6}x_{3}+\beta_{8}x_{1}+\beta_{9}x_{2}=0,$$

The minimum was calculated by solving the above equations. Validation was conducted by measuring IgG binding immunochemically and dimerization by polyacrylamide gel electrophoresis (SDS-PAGE) on the region surrounding the calculated minimum.

### 2.6. The Bicinchoninic Acid (BCA) Protein Assay

The protein contents were determined by a bicinchoninic acid kit (Sigma^®^) using bovine serum albumin (BSA) as a standard. BSA was diluted with 50 mM PBS (pH 7.4) to 0.8, 0.6, 0.4 and 0.2 mg mL^−1^. Either BSA standards or sample protein (25 μL), and streak reagent (200 μL) were incubated in duplicate in the respective wells in a microtiter plate at 37°C for 30 min. Absorbance was measured at 562 nm by a spectrometer (Molecular Devices SpectraMax M2).

### 2.7. SDS-PAGE

Protein samples (0.3 mg mL^−1^) were diluted with Laemli sample buffer (1:1 *v*/*v*), 20 μL/well, were applied onto 15 % polyacrylamide gel with 4% stacking gel. Before electrophoresis, protein samples were heated at 95 °C for 5 min. Precision Plus Protein™ Prestained Standards (BioRad^®^) ranging from 2 kDa to 250 kDa were used as molecular weight markers. Electrophoresis was conducted using a Mini Protean Tetra Cell (BioRad^®^) at a constant voltage of 150 V until the sample dye reached the end of the gels. The gels were stained with Coomassie brilliant blue R-250. Finally, the gels were scanned and quantified with the ChemiDoc MP System (Bio-Rad^®^).

### 2.8. Enzyme-Linked Immuno-Sorbent Assay (ELISA)

Rabbit anti-β-Lg, rabbit anti-α-La and goat anti-whey protein were generated according to the approved UNSW Sydney animal ethics (ACEC11/2A). For immunization, β-Lg and α-La were diluted to 0.25–1 mg mL^−1^ in 0.9% NaCl and mixed with an equal volume of Titer Max Gold adjuvant. Antisera containing specific antibodies were collected from New Zealand white rabbits (UNSW, Sydney, Australia) and goats (Monash University, Melbourne, Australia) following a strict immunization regime [40]. The IgG antibodies were purified from the sera using protein A affinity chromatography.

Two ELISAs each specific to β-Lg and α-La were developed and optimized. In this study, the antibody binding (%) measured by the ELISAs was used to determine the relative molecular changes induced by the HHP-thermal processing. In brief, β-Lg and α-La stock solutions at 10 mg mL^−1^ were prepared in phosphate-buffered saline (PBS, pH 7.4). Capture antibodies (anti-β-Lg antibody or anti-α-La antibody at 10 µg mL^−1^ in carbonate buffer, pH 9.6) were coated on a microtiter plate by overnight incubation. After washing with 0.05% Tween 20 in water, the plates were incubated for 1h with 1% soybean protein in PBS (50 mM phosphate buffer—0.9% sodium chloride). The microtitre plates were then washed at least three times with 0.05% Tween 20 in water. Standard solutions (or calibration solutions) of whey protein were prepared by 2-fold dilutions with the initial concentrations of 240 and 200 μg L^−1^, for β-Lg and α-La respectively. The plate was incubated for 30 min on a rotary shaker. After washing with 0.05% Tween 20 in water, a detection antibody (goat anti-whey protein antibody) was added to respective wells and incubated for 30 min. An anti-goat IgG antibody conjugated with horseradish peroxidase solution prepared in 1% bovine serum albumin (BSA) containing 0.05% Tween 20 in PBS was incubated for 30 min after unbound detection antibody was removed. The chromogenic substrate solution containing hydrogen peroxide and 3,3′4,4′-tetramethylbenzidine was incubated for 20 min after the final washing to allow for color development. The color reaction was stopped with 0.125 M sulfuric acid. The absorbance was measured at 450 nm using a SpectraMax M2 microplate reader (Molecular Devices). Immunoreactivity (defined as % relative IgG-binding) was calculated from the calibration curve of untreated protein.” IgG-binding (%) was calculated using the following expression:

(6) $$\mathrm{IgG}-\mathrm{Binding}\;\left(\%\right)=\frac{\mathrm{Detected}\;\mathrm{concentration}\;\mathrm{of}\;\mathrm{treated}\;\mathrm{sample}}{\mathrm{Concentration}\;\mathrm{of}\;\mathrm{untreated}\;\mathrm{sample}\;\left(\mathrm{control}\right)}\times 100,$$

### 2.9. Spectral Measurements

Circular dichroism (CD) spectra of HHP-thermal processed β-Lg at 0.1 mg mL^−1^ in 0.03 M phosphate buffer with 0.3% NaCl (pH 7.2) were recorded by a Chirascan™-plus CD Spectrometer (Applied Photophysics, Leatherhead, Surrey, UK), equipped with a Peltier temperature controller thermalstated at 25 °C. Sample cell paths were set for 0.5 mm (quartz cuvette), with a spectral resolution of 0.5 nm and averaging time of 0.5 s.

Fluorescence spectra were recorded using a Cary Eclipse fluorescence spectrometer (Agilent Technologies, Santa Clara, CA, USA at 25 °C. Protein solutions (0.1 mg/mL) were excited at 290 nm, and the emission was automatically recorded at right angles to the excitation with a 5-nm bandwidth in the range 300–400 nm.

### 2.10. Dot Blotting with Rabbit IgG and Human IgE

Seven serum samples from milk allergic patients with the confirmed clinical diagnosis of IgE-mediated milk allergy were provided by the Children’s Hospital at Westmead (Sydney, Australia) with human ethics approval #LNR/14/SCHN/516. All individuals (2 females and 5 males) had had a clinical reaction to cow’s milk and positive ssIgE. Milk-specific IgE ranged from 0.67 to greater than 100 kU/L (median 7.05). Six out of the 7 had a positive ssIgE within 12 months of their last reaction. Only one child (Patient 7), who had had anaphylaxis to cow’s milk (as their last reaction), but their ssIgE was done 3 years before their reaction and remained cow milk allergic at their last evaluation. Four individuals had a positive skin test to cow’s milk extract >3 mm. These samples were screened for the component-specific IgE by immunoblots (data not shown). The serum IgEs were reacted to one or more of the following milk components: β-Lg, α-La, BSA, IgG and casein. Two serum samples showing distinct IgE binding to β-Lg and α-La were selected for our study. The control serum was from a healthy volunteer.

Five HHP-thermal treated β-Lg with minimum IgG-responses were selected to assess their human IgE immunoreactivity as an indication for potential allergenic potential. These were; 600 MPa-25 °C-115 min, 80 °C-75 min, 600 MPa-65 °C-35 min, 400 MPa-45 °C-142.28 min and 400 MPa-78 °C-75 min. Immunoblots were prepared by loading 2 µL of 0.3 mg/mL β-Lg solution from each treatment in triplicates on 0.45 µm nitrocellulose membranes (BioRad^®^). The membrane was air-dried then blocking overnight with 1% soybean protein in PBS buffer. After washing, each blot was incubated with the selected rabbit IgG antibody diluted 1:2000 (for IgG) or patient serum in 1:20 in blocking solution overnight at room temperature with continuous shaking. The serum sample was selected based on its specificity to β-Lg in the screening. Serum from a nonallergic individual was used as a control. The blots were then vigorously washed thrice with PBS with 0.05% Tween 20, followed by incubating with mouse anti-human IgE Fc-HRP (Southern Biotech^®^, Birmingham, AL, USA) for 1h at room temperature. After washing, the signals were developed with SuperSignal™ West Dura Extended Duration Substrate (ThermoFisher Scientific^®^, Waltham, MA, USA) and read by the ChemiDoc MP System (Bio-Rad^®^).

### 2.11. Statistical Analysis

All the data were analyzed for statistical significance by t-test, one-way ANOVA followed by Tukey’s multiple comparisons test and 3-way-ANOVA with α = 0.05, using GraphPad Prism (GraphPad Software, LLC, San Diego, CA, USA) and Matlab (The MathWorks, Inc., xperiments were performed in triplicates and data were expressed as mean ± SD.

## 3. Results and Discussion

### 3.1. Preliminary Study of Pressure, Holding Time and IgG Binding

Before the HHP experiments were conducted, the protein concentration (i.e., 300 μg mL^−1^) was optimized for the HHP experiments to ensure the protein solubility was maintained and concentration was sufficient for the subsequent molecular analysis. At higher concentrations, protein aggregation was observed after the HHP in some samples and during the cold storage, which impacted the molecular analysis. The preliminary study of HHP parameters, pressure and holding time on the IgG-binding activity of β-Lg were conducted to determine the appropriate ranges of key operating parameters involving pressure and holding time required for a factorial experimental design.

**Figure 1:**
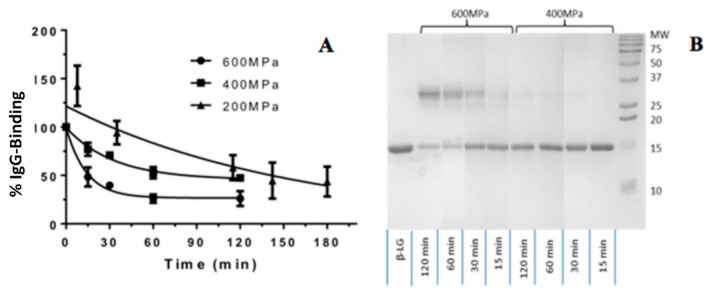
Preliminary study of IgG binding of HHP induced β-Lg (**A**) and SDS-PAGE of the protein profile of HHP induced protein modification (**B**).

Three pressures (200, 400 and 600 MPa) were selected to determine the working pressure range. The holding time ranged from 0–120 min for 400 MPa and 600 MPa and 0–180 min for 200 MPa. All the experiments were conducted at ambient temperature (25 °C). The results of preliminary study are shown in Figure 1. All the samples were evaluated by ELISA and SDS-PAGE for processing effects. The SDS-PAGE revealed dimerization of β-Lg as a distinct band at 37 kDa. Toggling between monomeric at 18 kDa and dimeric structures at 37 kDa in SDS-PAGE was more visible at 600 MPa than 400 Pa. The degree of β-Lg dimerization was influenced by the holding time. The IgG binding was significantly reduced from 0–30 min at 400 MPa and 600 MPa. The effects were then diminished after 30 min with % IgG binding of remining at 25%. A gradual reduction in % IgG bining at 200 MPa with increasing holding time was observed. Notably the standard deviation of % IgG binding at 200 Mpa was greater than those at higher pressures.

### 3.2. Effects of Process Variables on the Molecular Changes of β-Lactoglobulin Determined by IgG Binding

An antibody-antigen interaction is a specific phenomenon relying on an antibody’s ability to specifically bind to its antigen. Based on this, the HHP induced relative molecular changes of β-Lg with three processing variables (pressure, temperature and holding times) were determined by the IgG-reactivity. Before the ELISA, all the samples before and after the treatment were analyzed for protein concentration to ensure there was no loss of protein due to non-specific adsorption to the vial or aggregation and precipitation.

**Figure 2:**
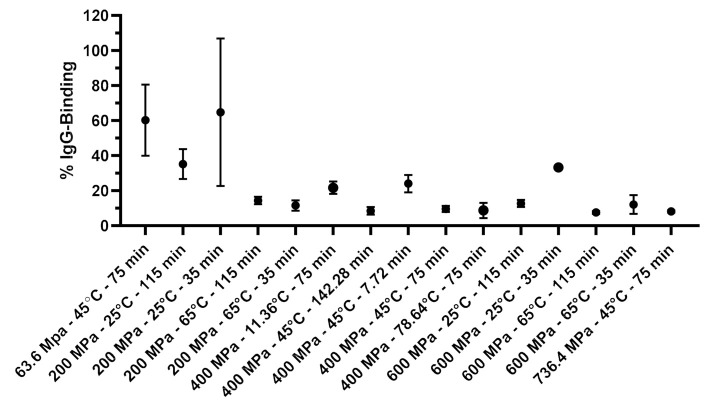
The %IgG binding of HHP treated β-Lg at different HHP conditions.

The % IgG-binding of 15 processing conditions are illustrated in Figure 2. The summary of one-way ANOVA and Tukey’s multiple comparisons test is shown in Supplementary Table S1. As in the preliminary study, lower pressures tended to induce variable % IgG binding with greater deviations (20–42.1%). The apparent anomalous increase in larger standard deviations were probably due to the large structural heterogeneity present in the molecular transition state. In Figure 2, 15 treatment samples were devided into two categories (a and b) based on their IgG binding activity (mean ± SD). Treatments with the same letters were statistically insignificant.

The treatments conducted at 400 MPa and above reduced the relative % IgG binding to less than 25% of the untreated β-Lg. The lowest IgG binding achieved in this system was 7.5% when β-Lg was treated at 600 MPa, 65 °C for 115 min. High pressure alone was less effective in reducing IgG binding of β-Lg than when high pressure was combined with moderate thermal treatment. Generally, raising treatment temperature decreased the IgG binding in all the combinations of pressure and holding time The effect of temperature was particularly noticeable at a holding time of 35 min, when the temperature was increased from 25 to 65 °C, showing an average decrease by 53.3% and 21.2% at 200 MPa, and 600 MPa respectively. At a longer holding time of 115 min, however, increasing temperature from 25 to 65 °C reduced the IgG-binding only by 20.8% and 5.2% at 200 MPa, and 600 MPa respectively.

Increasing pressure reduced IgG binding for all the temperature and time combinations. The IgG binding generally decreased with an increasing holding time. The effect of holding time was more evident at 25 °C; at a lower temperature, a longer holding time was needed to reduce the IgG binding. The treatment at 200 MPa and 65 °C did not show a further reduction in the IgG binding when the holding time was increased from 35 to 115 min (Figure 2). This indicated that the protein modification occurred more rapidly during the first few minutes of the treatment.

All the treatments except for that at a lower temperature and shorter holding time (25 °C and 35 min) or lower pressure and lower temperature (i.e., 200 MPa and 25 °C) were able to reduce the IgG binding below 15%.

In this work, the IgG binding of β-Lg was greatly influenced by pressure and temperature indicating the susceptibility of β-Lg structure to high pressure. The molecular susceptibility of β-Lg to HHP was also supported by other studies [41,42]. For instance, the treatment at 600 MPa and 50 °C, for the selected times up to 64 min, induced a conformation change of β-Lg [41]. Karamanova et al., [42] also claimed changes in β-Lg could start at pressures as low as 100 MPa. At 200 MPa, formations of β-Lg non-native monomers and dimers were observed. At pressures beyond 500 MPa, β-Lg denatures extensively and aggregates to form polymers. Likewise, longer treatment times increase HP-induced denaturation and aggregation [42]. In our study, formation of dimers was not observed until 400 MPa, possibly due to the low protein concentration (Figure 1).

### 3.3. The CD and Intrinsic Fluorescence Analyses of β-Lg Treated with Selected HHP Conditions

**Figure 3:**
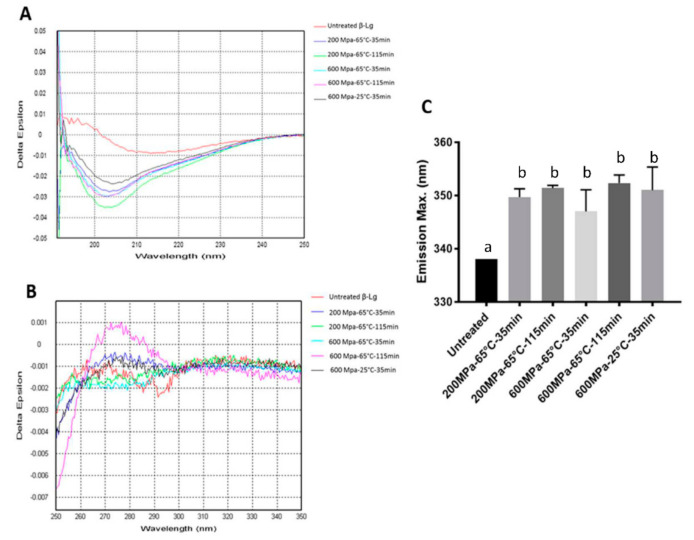
The CD spectra and fluorescence emission of untreated and HHP treated β-Lg: (**A**) Far-UV CD spectra, (**B**) near-UV CD spectra and (**C**) emission maxima of tryptophan fluorescence. Triplicate samples were used. Treatments with the same letters are statistically insignificant.

In this study, the CD and intrinsic fluorescence analyses were performed for five selected operating conditions with lower IgG-binding to analyze the secondary and tertiary protein structure modification (Figure 3). The low protein concentration used for the study (300 μg mL^−1^) was not ideal for the CD analysis, hence the quality of CD generated was not of high quality. Nevertheless, it was adequate to provide minimum information regarding the molecular changes.

The increased intensity of the trough at 205 nm was found in the far-UV CD spectra indicating that the *β*-sheet content was essentially reduced (Figure 3). The shifting could be a consequence of the decreased ratio between the native monomers and induced dimers in the mixture (SDS-PAGE) and the loss of H-bonding involving the I-strands of β-Lg [43].

In the far-UV CD (Figure 3A), the trough, with a minimum at 216 nm, shifted to lower wavelengths. The increased intensity of the trough at 205 nm in the spectra supported that the β-sheet content was essentially reduced by processing. The shifting could be a consequence of the decreased ratio of native monomers to dimers in the mixture and the loss of H-bonding involving the I-strands of β-Lg. In the near-UV CD spectra (Figure 3B), there was a reduction in the depth of two troughs at 286 nm and 293 nm, suggesting that some ordered tertiary structure was no longer present in the proteins after HHP processing. Also, the spectra showed a significant and reproducible difference in the 250–270 nm regions, which corresponded to the disulfide bonds of Cys^66^–Cys^160^ and Cys^106^–Cys^119^. The increasing intensity of the 250–270 nm region suggested the involvement of disulfide bonds of the processed β-Lg in the molecular changes.

Intrinsic fluorescence results (Figure 3C) were in the agreement with the CD spectra. The A right shift of emission spectra with a shift of λ_max_ from 338 to 347–352 were observed. There was a shift of 9–14.4 nm in the emission spectra, indicating the unfolding of β-Lg and the exposure of tryptophan residue as a result of tertiary structure changes. There was no significant difference between the degree of protein unfolding in the five processing conditions. However, it was apparent that the level of pressure applied to the protein influenced the degree of protein unfolding (10.3 ± 1.9 nm at 200 MPa vs 13.6 ± 0.7 nm at 600 MPa). The modification of protein structure evidenced by the CD and intrinsic fluorescence analyses, therefore, supported the changes observed with the IgG binding.

Our results of reduced IgG binding induced by HHP-thermal processing, however, opposed the results by Kleber et al. [17] and Meng et al. [44] who reported that the IgG binding of β-Lg increased with increasing pressure and holding time from 200 to 600 MPa for up to 30 min. They also found that increasing temperature from 30–68 °C resulted in increasing IgG reactivity. This difference could be due to the different sample matrix effects, for which Kleber et al. used milk while our results were based on purified β-Lg. Another possible reason would be the monoclonal antibody (IgY) they used could recognize linear epitopes in unfolded structures, resulting in the increased antibody binding. Whereas our polyclonal antibodies were raised against the native protein and were likely to be more sensitive to molecular changes from the native form (i.e., loss of IgG binding).

### 3.4. CCD of IgG Binding of HHP Treated β-Lactoglobulin

**Figure 4:**
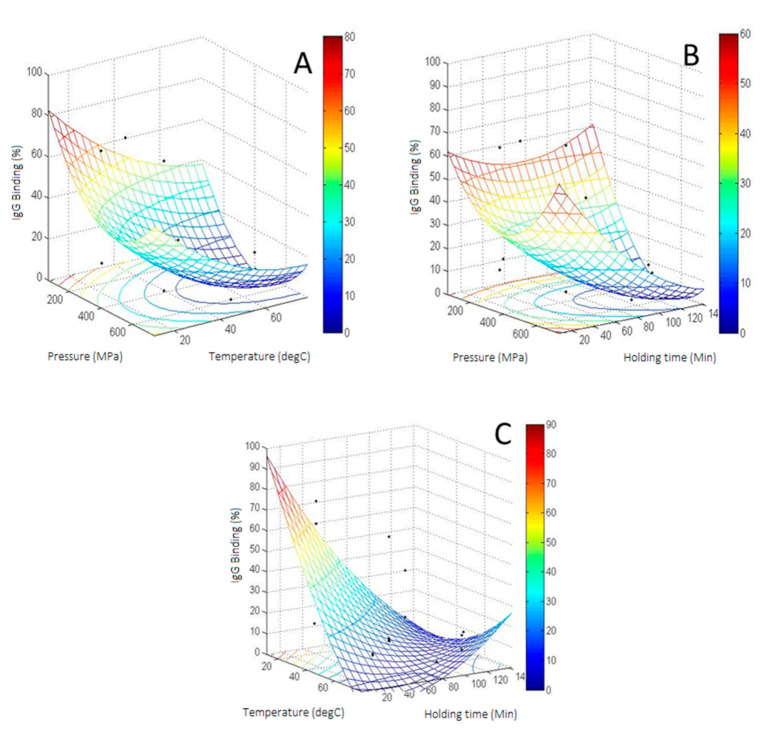
Surface plot of the combined effects of (**A**) pressure and temperature; (**B**) pressure and holding time; (**C**) temperature and holding time on the % IgG binding of HHP-Heat treated β-Lg.

The response surface plots displayed the interaction effects of pressure and temperature at a constant holding time of 75 min (Figure 4A), pressure and holding time at a constant temperature of 45 °C (Figure 4B), and temperature and holding time at 400 MPa (experimental values are displayed as black points, Figure 4C). Increasing pressure and temperature resulted in a minimum area of molecular changes determined by the IgG binding (Figure 4A). The experimental values on the minimum area were placed above the surface plot, indicating that the model may have underestimated the molecular changes. The experimental IgG binding did not decrease below 7.5% despite the calculations suggesting otherwise. Likewise, the IgG binding area was lowest for the pressures between 400–600 MPa and holding times between 75–115 min (Figure 4B). Figure 3C showed the cumulative reduction of IgG binding when higher temperatures and longer holding times were combined (Figure 4C).

Average IgG binding data obtained experimentally was fitted into a second-order model yielding an R^2^ value of 0.81. The model was able to explain over 81% of the variability in the β-Lg IgG reactivity. The equation is shown below:

(7) $$\begin{array}{c} y=-0.2x_{1}-2.495x_{2}-0.956x_{3}+0.000253x_{1}^{2}+0.008x_{2}^{2}+0.002x_{3}^{2}+0.000167x_{1}x_{2}-0.001x_{1}x_{3}+0.014x_{2}x_{3}+170.941,\\ \left(x_{1}=\mathrm{Pressure}\;(\mathrm{MPa});\;x_{2}=\mathrm{Temperature}\;(\text{°}C);\;x_{3}=\mathrm{Time}\;(\min)\right) \end{array}$$

The R^2^ value can be considered as adequate for such biological response characterized by diverse protein transitions upon minimal or moderate processing conditions. Analysis of variance (3-way ANOVA) also showed that the model was significant with a *p*-value of 0.01 (Supplementary Table S2). The model showed the significant effect of temperature (linear effect), pressure (quadratic effect) and the interaction between temperature and time. Coefficients of the model for temperature, pressure^2^ and the temperature by time interaction were significant with *p*-values smaller than 0.05 (Supplementary Table S2). Other coefficients were insignificant, indicating only limited effects on the molecular changes.

### 3.5. CCD of Dimerization of HHP Treated β-Lg

**Figure 5:**
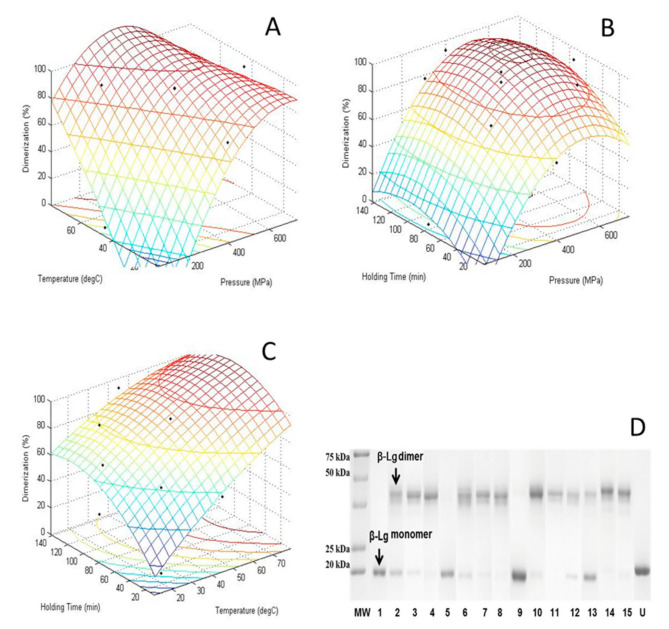
Surface plot of the combined effects of (**A**) pressure and temperature; (**B**) pressure and holding time; (**C**) temperature and holding time on the dimerization (%) of HHP treated β-Lg; (**D**) non-reducing SDS-PAGE analysis of HHP-Heat treated β-Lg ((1) 200 MPa-25 °C-35 min, (2) 600 MPa-25 °C-35 min, (3) 200 MPa-65 °C-35 min, (4) 600 MPa-65 °C-35 min, (5) 200 MPa-25 °C-115 min, (6) 600 MPa-25 °C-115 min, (7) 200 MPa-65 °C-115 min, (8) 600 MPa-65 °C-115 min, (9) 63.6 MPa-45 °C-75 min, (10) 736.4 MPa-45 °C-75 min, (11) 400 MPa-11.36 °C-75 min, (12) 400 MPa-78.64 °C-75 min, (13) 400 MPa-45 °C-7.72 min, (14) 400 MPa-45 °C-142.28 min, (15) 400 MPa-45 °C-75 min and (U) Untreated).

The SDS-PAGE of HHP-thermal β-Lg, at the levels of variables listed in Table 1, under non-reduced conditions are shown in Figure 5D. The formation of non-native dimers (~30.4 kDa) from their monomers (~15.9 kDa) was obvious. HHP-thermal induced β-Lg dimer was quantified from SDS-PAGE using ChemiDoc MP system (Biorad). The %Dimer and %Monomer were calculated based on their band intensity. The degree of dimers formed was influenced by the treatment conditions. For example, no dimers were formed when β-Lg was treated at 200 MPa and 25 °C for 35 min. Small amounts of dimers (<20%) were formed at low pressure and temperature, such as 200 MPa, 25 °C and 115 min and 63.6 MPa, 45 °C and 75 min, while more than 50% of the native monomers converted to dimers in most other treatment conditions.

The formation of dimers from the native monomeric β-Lg has been reported by [45]. According to Considine et al. [46], the application of pressure results in various events such as protein unfolding, exposure of internal free sulfhydryl groups, disulfide bond interchanges and aggregation. Upon depressurization, exposed groups could react with free S-H groups. Those groups may not return to their native forms before the protein refolds. Pressure may also induce differential charge distribution. Slight differences in charge distribution may cause two molecules to interact forming a stable dimer if conformational changes enable appropriate groups on the surface to interact with each other [45]. In our study, pressure above 400 MPa induces more than 90% of dimerization. This is expected because the likelihood of two molecules interacting to form stable dimers increases at higher pressure and temperature.

A regression analysis (Supplementary Table S3) was carried out to fit the dimerization of HHP treated β-Lg as a function of the process variables, pressure, temperature and holding time. The model is described by the equation below:

(8) $$\begin{array}{c} y=20.99x_{1}+17.54x_{2}+7.87x_{3}-15.09x_{1}^{2}-0.43x_{2}^{2}-3.59x_{3}^{2}-16.05x_{1}x_{2}-2.37x_{1}x_{3}-6.82x_{2}x_{3}+92.72,\\ \left(x_{1}=\mathrm{Pressure}\;(\mathrm{MPa});\;x_{2}=\mathrm{Temperature}\;(\text{°}C);\;x_{3}=\mathrm{Time}\;(\min)\right) \end{array}$$

The significance of each coefficient was determined by the 3-way ANOVA (Supplementary Table S3). The variables with the largest effect on the dimerization were the linear terms of pressure and temperature, the quadratic term of pressure, and the interaction between pressure and temperature. The pressure and temperature had highly significant effects on dimerization (*p* < 0.001) while holding time had a less significant effect (*p* = 0.0506). The coefficient of determination (R^2^) of the model was 0.93 showing a good fit.

Figure 5A–C shows the response surface plots of dimerization as a function of temperature and pressure at a constant holding time of 75 min (Figure 5A), a function of holding time and pressure at a constant temperature of 45 °C (Figure 5B), and a function of holding time and temperature at constant pressure of 400 MPa (Figure 5C). The dimerization increased as both pressure and temperature increased (Figure 5C). An increase in dimerization was mainly influenced by increasing temperature and/or pressure, while holding time had little influence (Figure 5B,C).

### 3.6. Relationship between IgG Binding and Dimerization

**Figure 6:**
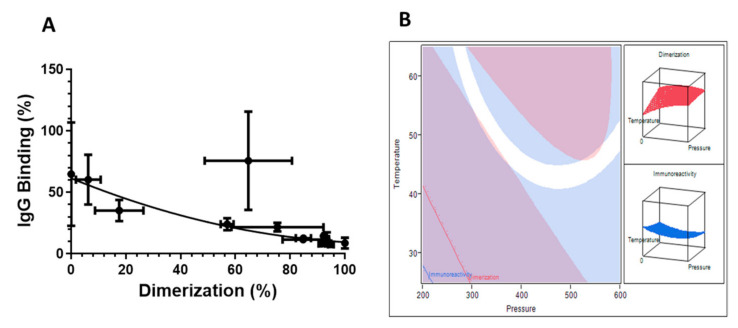
The plot of dimerization vs the % IgG-binding of β-Lg. (**A**) Equation: y = 61.48 − 0.9103x + 0.003909x^2^, R^2^ = 0.9482) and (**B**) Overlay plot of % IgG-binding and dimerization of β-Lg in the constraint setting (Blue area: % Antigenicity > 10%; Pink area: % Dimerization < 90%).

The relationship between IgG binding and dimerization of β-Lg is illustrated in Figure 6. The dimerization resulted in lower IgG binding, which followed the quadratic equation in Figure 4. The equation showed a good correlation between the reduced IgG binding and the increased dimerization, exhibiting an R^2^ value of 0.95. There was one point that showed large deviations in both directions. The processing condition of the point was at the lower level for the three process variables, at 200 MPa, 25 °C and 35 min of holding time. At this intermediate condition, it is expected that the protein undergoes diverse transitional states, hence showing a greater heterogeneity in the IgG binding. Transitional modification is not always consistent for every protein molecule and this exacerbates at the intermediate process conditions. The standard deviations of protein transition in the other treatments were within 5%, indicating a more consistent and reproducible protein transition at those higher conditions. Figure 5 shows two overlapping contour plots of dimerization (pink color) and IgG binding (blue color). The overlay area is displayed in white color by setting IgG binding between 7.5–10% and dimerization between 90–100%. The overlapping region of low IgG binding (7.5–10%) and high dimerization (90–100%) occurs at a pressure between 400–600 MPa and temperatures between 40–50 °C at a constant holding time of 75 min. Reducing pressure to the range of 300–400 MPa would require a higher temperature between 50 to 65 °C to keep it within the overlapping region.

Considering the immunogenic epitopes on β-Lg, the coexistence of high dimerization and low IgG binding can be explained by either masking or modifying conformational epitope moieties upon dimerization that becomes less accessible to the antibody [47]. HHP, as a powerful process, generates folding conformational states of β-Lg [48]. High pressure either as single processing or combined with thermal processing promotes transient modifications of the β-Lg structure at neutral pH, inducing transient monomer to dimer, burying the previously exposed hydrophobic sites. The CD and instinct fluorescence measurements demonstrated the irreversible effects of high-pressure treatment on the secondary and tertiary structures of β-Lg.

### 3.7. Determination and Validation of Calculated Optimum Response

The optimal conditions derived from the model were 505 MPa of pressure; a temperature of 56 °C and a holding time of 102 min. Under these conditions, the % IgG binding (i.e., molecular changes) calculated with Equation (7) was 1.4%. A supplementary experiment was carried at the optimal conditions and its surrounding region as a validation (Table 2). The experimental IgG binding remained between 20–35% with 70–85% of dimerization. The experimental results disagreed with the calculated values, suggesting that the model indeed underestimated the IgG-binding around the minimum area. Nevertheless, the correlation between IgG binding and dimerization displayed in Figure 5 statistically fits better than the equation generated by RSM when the exclusion of the point with high standard deviation was applied (columns 5 and 6 on Table 2).

### 3.8. Effects of Thermal and HHP Treatments on α-La

The HHP-thermal-induced α-La IgG binding ranged from 52 to 144% compared to the untreated α-La. The standard deviations were considerably large ranging from 8 to 83%. Due to the large standard deviations, the ANOVA shows no significant effects in all of the tested process parameters (model *p*-value of 0.34) (Supplementary Table S4). With an R^2^ value of 0.54, the model did not correlate the IgG binding response with any of the process variables. The regression coefficients of most parameters were insignificant. Only the pressure was found to be significant with a *p*-value of 0.05.

There were no aggregates detected in all the HHP treated, HHP-thermal combined and the thermal-only treated α-La. The large standard deviation observed in this study was likely to be attributed to the reversible denaturation or refolding of α-La molecule since α-La had no free sulfhydryl groups for dimerization to occur readily [49,50,51,52]. As proteins undergo structural modification to new conformational states upon HHP-thermal processing, previously hidden linear or conformational epitopes could be revealed for IgG binding. The large standard deviations thus support this proposition as refolding occurs at a molecular level but does not replicate analogously across all molecules. Nevertheless, due to the random nature of α-La IgG binding, the fitted model and its related contour plots were disregarded.

### 3.9. Dot-Blotting with Rabbit IgG and Human IgE

Roughly 60% of adverse reactions to cow’s milk are related to IgE-mediated allergy and human IgE responses to cow’s milk proteins are characterized by a diverse specificity and sensitivity [53,54,55]. Our pool of sera from the patients with the confirmed clinical diagnosis of IgE-mediated milk allergy also showed a diverse IgE-binding signature. Due to the limited number and supply of the available patient sera with specific binding to β-Lg and α-La, only two serum samples each with distinct IgE binding to β-Lg and α-La were selected to determine the IgE-binding to HHP-treated proteins. Here, we compared the rabbit IgG-reactivity, as the relative molecular changes, to that of human IgE-reactivity.

**Figure 7:**
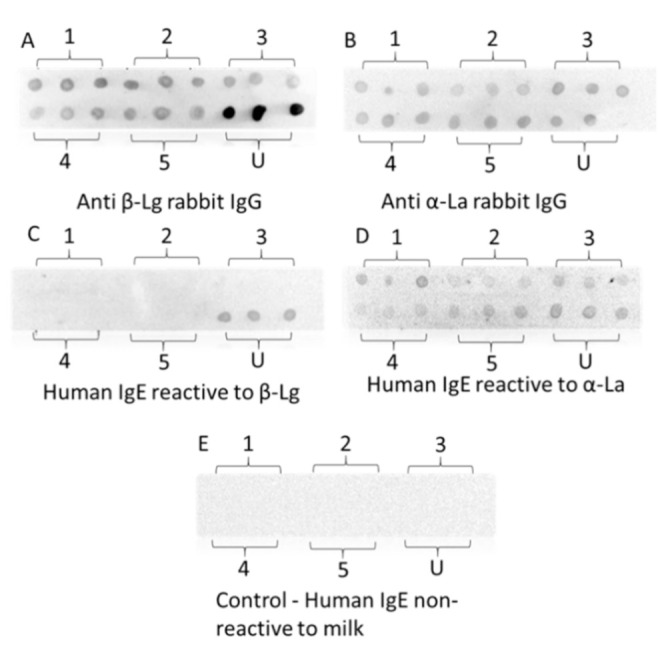
Dot blots of untreated and HHP-heat-processed β-Lg and α-La with anti-β-Lg rabbit IgG (**A**), anti- α-La rabbit IgG (**B**) and human IgE (**C**,**D**). (**E**) Control dot bot with serum from a non-allergic individual. 1–6 represented different treatments: 1. 600 MPa-25 °C-35 min; 2. 80 °C-75 min; 3. 600 MPa-65 °C-35 min; 4. 400 MPa-45 °C-142.28 min; 5. 400 MPa-45 °C-75 min; U. Untreated.

As shown in Figure 7, all five treatments demonstrated a similar significant reduction in the rabbit IgG binding. The decreased rabbit IgG binding indicated that treatment-induced protein structural modification through the disulfide bond and hydrophobic interchange between the proteins [56,57] and dimerization (Figure 5) led to a loss of IgG conformational epitopes. Very promisingly, the treated β-Lg showed undetectable human IgE binding in the dot blots, showing the potential of using HHP-thermal processing to produce dairy-based products with lower IgE-reactivity. As aforementioned that milk allergic sera show diverse IgE binding profiles to different milk allergens, it is important to confirm the reduced IgE binding with more patient serum samples.

On the other hand, there was an unnoticeable change in both rabbit IgG and human IgE binding for α-La after the treatment at five different conditions. These results were consistent with some earlier studies which showed that α-La is highly resistant to HHP due to the presence of four disulfide bonds in the ternary arrangement, creating structural rigidity [46,57,58]. Additionally, there are moderately weak hydrophobic interactions between unfolded α-La resulting in high thermal resistance [59,60]. These findings consolidate the effects of HHP-thermal processing to be beneficial for producing hypoallergenic dairy products.

## 4. Conclusions

A response surface methodology with a central composite design was conducted to study the effects of pressure, temperature and holding time on the molecular changes as determined by the rabbit IgG binding and dimerization of β-Lg, and α-La. The model showed the significant effect of temperature, the square of pressure and the interaction between temperature and time, but it underpredicted the minimum value. The β-Lg dimerization is significantly affected by pressure, temperature, the square of pressure and the interaction between pressure and temperature. The ANOVA of α-La IgG binding showed no or little significant effects of temperature, pressure or holding time, suggesting that α-La as a single protein is more tolerable to high pressure This study revealed for the first time that β-Lg dimerization resulted in lower IgG binding which followed a quadratic equation with an R^2^ value of 0.95.

The RSM was shown to be an effective method to correlate and optimize the effects of the HHP process variables on biological responses. The methodology proved to be adequate for the design of a process yielding a significant molecular change with minimum IgE binding against β-Lg. The negligible residual IgE-reactivity of HHP treated β-Lg demonstrates a promising potential of high-pressure processing to lower the allergenic potential of whey-based products while adding value to its growing market. As only a limited number of milk allergic sera was used as a proof of IgE-binding, hypoallergenicity should be further confirmed with additional representative serum samples and other means such as basophil activation assay. Also, further study on more complex milk or whey matrices containing both β-Lg and α-La is needed to provide greater insights into how these molecules interact with each other and behave under high-pressure conditions.

## Figures and Tables

**Table 1:** Experimental range and levels of the independent variables.

	Code-Level of Variables			Experimental Level of Variables		
Test Number	x_1_	x_2_	x_3_	P (MPa)	T (°C)	t (min)
1	−1	−1	−1	200	25	35
2	+1	−1	−1	600	25	35
3	−1	+1	−1	200	65	35
4	+1	+1	−1	600	65	35
5	−1	−1	+1	200	25	115
6	+1	−1	+1	600	25	115
7	−1	+1	+1	200	65	115
8	+1	+1	+1	600	65	115
9	−1.68	0	0	63.6	45	75
10	+1.68	0	0	736.4	45	75
11	0	−1.68	0	400	11.36	75
12	0	+1.68	0	400	78.64	75
13	0	0	−1.68	400	45	7.72
14	0	0	+1.68	400	45	142.28
15	0	0	0	400	45	75
16	0	0	0	400	45	75
17	0	0	0	400	45	75
18	0	0	0	400	45	75
19	0	0	0	400	45	75
20	0	0	0	400	45	75

x_1_ = Pressure (MPa); x_2_ = Temperature (°C); x_3_ = Time (min).

**Table 2:** Model validations.

	Variables			%IgG Binding	Dimerization	% IgG Binding	% IgG Binding
Test Number	P (MPa)	T (°C)	t (min)	Exp. Value	Exp. Value	Cal. Value ^a^	Cal. Value ^b^
1	505	56	102	21.4 ± 6.4	84.6	1.4	12.6
2	505	51	102	27.2 ± 0.5	71.2	1.6	16.5
3	505	61	102	29.2 ± 0.6	74.9	1.5	15.2
4	455	56	102	19.4 ± 1.8	74.9	2.1	15.2
5	555	56	102	32.6 ± 7.3	76.4	2.0	14.8
6	505	56	92	34.5 ± 8.6	71.5	1.7	16.4
7	505	56	112	27.4 ± 12.5	75.4	1.6	15.1

^a^ Calculated value using equation 3 from RSM. ^b^ Calculated value using the equation from Figure 4A.

## Supplementary Materials

The following are available online at https://www.mdpi.com/article/10.3390/foods10081741/s1, Table S1: One-way ANOVA analysis and Tukey’s multiple comparisons test of IgG binding of β-Lg treated with 15 different HHP conditions. Only statically significant pairs are listed., Table S2: Three-way ANOVA analysis of CCD of IgG binding of HHP treated β-Lg., Table S3: The 3-way ANOVA of the response surface model of β-Lg dimerization. Table S4: Three-way ANOVA of CCD of IgG binding of HHP treated α-La.
