# Supplementary material for: A Response Surface Methodology (RSM) Approach for Optimizing the Attenuation of Human IgE-Reactivity to β-Lactoglobulin (β-Lg) by Hydrostatic High Pressure Processing
Source: Foods. 2021 Jul 28;10(8):1741. doi: 10.3390/foods10081741 (PMC8394912; doi:10.3390/foods10081741)
Supplement: Supplementary file 1 [file foods-10-01741-s001.zip › foods-1272151-supplementary.pdf]

Supplementary Materials

Table S1. One-way ANOVA analysis and Tukey's multiple comparisons test of IgG binding of β-Lg treated with 15 different HHP conditions. Only statically significant pairs are listed.

	df	SS	MS	F
Treatment (between treatments)	14	16081	1149	10.12
Residual (within treatments)	42	4769	113.5	Significance F
Total	56	20850		<0.0001

	Mean diff.	q	Adj.Pvalue
T1 vs. T4	45.9	7.46	0.0004
T1 vs. T5	48.8	7.93	0.0001
T1 vs. T6	38.6	6.27	0.005
T1 vs. T7	51.8	8.41	<0.0001
T1 vs. T8	36.3	5.90	0.0108
T1 vs. T9	50.7	10.64	<0.0001
T1 vs. T10	51.5	8.38	<0.0001
T1 vs. T11	47.6	7.73	0.0002
T1 vs. T13	52.7	8.57	<0.0001
T1 vs. T14	48.2	7.83	0.0002
T1 vs. T15	52.1	8.46	<0.0001
T2 vs. T9	25.6	5.38	0.0297
T3 vs. T4	50.4	8.19	<0.0001
T3 vs. T5	53.2	8.65	<0.0001
T3 vs. T6	43.1	7.00	0.0011
T3 vs. T7	56.2	9.14	<0.0001
T3 vs. T8	40.7	6.62	0.0024
T3 vs. T9	55.2	11.57	<0.0001
T3 vs. T10	56.0	9.10	<0.0001
T3 vs. T11	52.0	8.46	<0.0001
T3 vs. T12	31.4	5.11	0.0484
T3 vs. T13	57.2	9.29	<0.0001
T3 vs. T14	52.6	8.56	<0.0001
T3 vs. T15	56.5	9.19	<0.0001

T1: 63.6 MPa - 45 degC - 75 min; T2: 200 MPa - 25 degC - 115 min; T3: 200 MPa - 25 degC - 35 min; T4:200 MPa - 65 degC - 115 min; T5: 200 MPa - 65 degC - 35 min; T6: 400 MPa - 11.36 degC - 75 min; T7: 400 MPa - 45 degC - 142.28 min; T8: 400 MPa - 45 degC - 7.72 min; T9: 400 MPa - 45 degC - 75 min; T10: 400 MPa - 78.64 degC - 75 min; T11: 600 MPa - 25 degC - 115 min; T12: 600 MPa - 25 degC - 35 min; T13: 600 MPa - 65 degC - 115 min; T14: 600 MPa - 65 degC - 35 min; T15: 736.4 MPa - 45 degC - 75 min.

Table S2. Three-way ANOVA of CCD of IgG binding of HHP treated β-Lg.

	df	SS	MS	F
Regression	9	6748.36	749.2	4.61
Residual	10	1627.56	162.6	Significance F
Total	19	8375.92		0.013
	Coefficients	Standard Error	t-Stat	P-value
Intercept	1.71E+02	41.61	4.11	0.00
Pressure (MPa)	-2.00E-01	0.10	-2.09	0.06
Temperature (°C)	-2.49E+00	0.99	-2.52	0.03
Time (min)	-9.56E-01	0.47	-2.03	0.07
$Pressure^2$	2.53E-04	0.00	3.01	0.01
$Temperature^2$	8.45E-03	0.01	1.01	0.34
$Time^2$	2.34E-03	0.00	1.12	0.29
Pressure x Temp	1.67E-04	0.00	0.15	0.89
Pressure x Time	-6.38E-04	0.00	-1.13	0.28
Temperature x Time	1.42E-02	0.01	2.52	0.03

Table S3. Three-way ANOVA of the response surface model of β-Lg dimerization.

	df	SS	MS	F
Regression	11	17108.30	1555.30	9.72
Residual	8	1279.87	159.98	Significance F
Total	19	18388.17		0.0017
	Coefficients	Standard Error	t Stat	*P*-value
Intercept	92.72	5.16	17.96	<0.0001
Pressure (MPa)	20.99	3.42	6.13	0.0003
Temperature (°C)	17.54	3.42	5.12	0.0009
Time (min)	7.87	3.42	2.30	0.0506
$Pressure^2$	-15.09	4.47	-3.37	0.0097
$Temperature^2$	-0.43	4.47	-0.10	0.9252
$Time^2$	-3.59	4.47	-0.80	0.4455
Pressure xTemp	-16.05	3.33	-4.82	0.0013
Pressure xTime	-2.37	3.33	-0.71	0.4968
Temperature xTime	-6.82	3.33	-2.05	0.0748

Table S4. Three-way ANOVA of CCD of IgG binding of HHP treated α-La.

	df	SS	MS	F
Regression	9	5306.95	589.66	1.31
Residual	10	4488.02	448.80	Significance F
Total	19	9794.98		0.337
	Coefficients	Standard Error	t Stat	P-value
Intercept	-5.06E+01	69.09	-0.73	0.48
Pressure (MPa)	2.99E-01	0.16	1.88	0.09
Temperature (°C)	3.32E+00	1.65	2.02	0.07
Time (min)	6.55E-01	0.78	0.84	0.42
Pressure^2	-3.12E-04	0.00	-2.24	0.05
Temperature^2	-2.34E-02	0.01	-1.68	0.12
Time^2	-3.59E-03	0.00	-1.03	0.33
Pressure x Temp	-8.52E-04	0.00	-0.45	0.66
Pressure x Time	1.77E-04	0.00	0.19	0.85
Temperature x Time	-8.30E-03	0.01	-0.89	0.40
